# The Potential of Condiments, Seasonings, and Bouillon Cubes to Deliver Essential Micronutrients in Asia: Scenario Analyses of Iodine and Iron Fortification

**DOI:** 10.3390/nu15030616

**Published:** 2023-01-25

**Authors:** Ans Eilander, Marieke R. Verbakel, Mariska Dötsch-Klerk

**Affiliations:** Unilever Foods Innovation Centre, 6708 WH Wageningen, The Netherlands

**Keywords:** modeling, fortification, iodine, iron, bouillon cubes, seasonings, condiments, intake/consumption, Asia

## Abstract

Micronutrient deficiencies are still highly prevalent in Asia. Fortification of cooking aids, such as condiments (fish and soy sauces), seasonings, and bouillon cubes, may be an additional strategy to improve micronutrient intake. The current study evaluated the potential impact of iodine and iron fortification of cooking aids on micronutrient intake in Asian countries. A systematic literature search was performed to collect consumption data from different countries in Asia. Data from 18 studies in nine Asian countries were included. Scenario analyses were performed using different fortification levels based on regulations and literature. Mean intake of cooking aids ranged from 3.2–15.9 g/day for condiments and 0.4–11.7 g/day for seasonings and bouillon cubes. When replacing salt with iodized salt (30 µg of iodine/g of salt), iodine intake would increase by 13–119 µg/day for soy and fish sauces (9–80% of the Nutrient Reference Value (NRV)), and 5–83 µg/day for bouillon cubes and seasonings (4–56% of the NRV). Fortification with iron 0.5 mg/g food product for condiments or 1 mg/g food product for bouillon cubes and seasonings improved iron intake for soy and fish sauces by 1.6–8.0 mg/day (11–57% of the NRV), and for bouillon cubes and seasonings by 0.4–5.6 mg/day (3–40% of the NRV). These results indicate that, depending on the consumption pattern, fortification of cooking aids can be a suitable strategy to increase intake of micronutrients.

## 1. Introduction

Deficiencies for vitamin A, iron, iodine, zinc, thiamin, and calcium are still widely prevalent in Asian countries [1]. Recently, it was estimated that 58% of preschool children and 72% of women of reproductive in the East Asia and Pacific region have at least one micronutrient deficiency [2]. Micronutrient deficiencies have severe health consequences and contribute significantly to the global burden of disease (GBD, 2019). In particular, iodine and iron deficiency have a large impact on the healthy growth and development of children.

Iodine is an essential trace element and important for the production of thyroid hormones. These hormones are important for fetal development, metabolic activities of cells, and growth and development of children [3]. Since 1995, the WHO recommends universal salt iodization, which includes the use of iodized salt not only at the table but also in food production [4]. While iodization of table salt is mandatory in most Asian countries, using iodized salt in processed foods is mostly voluntary [5]. However, due to dietary shifts toward increased consumption of processed foods in the region, universal iodization becomes increasingly important [6,7]. 

Iron is a major component of hemoglobin and plays an important role in oxygen transportation throughout the body. Deficiencies can lead to anemia, fatigue, developmental delay, cognitive impairments, and adverse pregnancy outcomes. Children and women of reproductive age are most at risk of inadequate iron intake due to high requirements for growth, menstruation, or pregnancy [8]. 

Food fortification, including fortification with iron and iodine, is one of the most cost-effective interventions from a global public health perspective, which does not require the consumer to change their dietary pattern [9,10]. Beyond fortification of staple foods (e.g., flour, salt, and oil), fortification of alternative products such as frequently used tastemakers, such as condiments, seasonings, and bouillon cubes, has been suggested as an additional strategy to improve micronutrient intake [11]. Cooking aids are widely consumed in high-, low-, and middle-income populations. They are affordable to people living in remote areas where most staple foods that are produced locally are unfortified [11]. Moreover, the efficacy of fortified cooking aids on improving micronutrient intake has been demonstrated in several trials with iron-fortified fish sauce in Vietnam [12], iron-fortified soy sauce in China [12], and double-fortified salt with iron and iodine in Morocco [13]. However, data on the efficacy of fortification of other cooking aids, such as seasonings and bouillon cubes, are rather limited. While a few modeling studies have been conducted to assess the potential impact of bouillon cube fortification on micronutrient intake in Africa [14,15] and Asia [16,17], available data from different countries on the potential impact of fortification are still limited.

Therefore, in the current study, a literature review was performed to evaluate the consumption patterns of different cooking aids focusing on Asia with the aim of performing scenario analyses simulating fortification with iodine and iron to evaluate the potential impact on micronutrient intake in the general adult population.

## 2. Materials and Methods

### 2.1. Search Strategy

A systematic literature search was conducted in Scopus, to find relevant studies with data on bouillon cubes, seasoning, and condiment consumption in Asian countries. A combination of the following terms was used to search abstracts, titles, and keywords: (condiment* OR seasoning* OR (bouillon* AND cube*) OR sauce* OR spice* OR (cooking AND aid*)) AND (consum* OR intake*).

Filters, including human species, English language, and Asian or “undefined” countries, were applied to restrict results to relevant publications. The results from the literature search were exported to Endnote. Furthermore, websites of public health organizations in Asia and reference lists of publications found were checked for additional studies. 

### 2.2. Inclusion and Exclusion Criteria

Articles were selected for full-text review if they included intake data from national surveys, population-based observational (cross-sectional or longitudinal) studies, or baseline or control group data from intervention studies. Subsequently, full-text articles were screened to determine eligibility on the basis of the following inclusion criteria:

1. National and subnational surveys or studies reporting quantitative data on either consumption of cooking aids or sodium/salt intake from these cooking aids, at individual or household level. Cooking aids were defined as condiments, including fish and soy sauces, bouillon cubes, bouillon powders, and seasoning products.
2. Studies and surveys conducted and published after the year 2000.

### 2.3. Data Extraction

Information on intake of condiments, seasonings, and bouillon cubes for adults was extracted as reported. Household consumption data were also included after conversion to individual intake level, by dividing household consumption by the average household size (mentioned in the paper) for that specific country. Where needed, weekly consumption was converted to daily intake. In addition, data that were reported separately for men and women were pooled into a mean value for both genders. Similarly, separately reported data for rural and urban areas were pooled using the ratio urban versus rural in the country in the year(s) the data were collected [18]. If the year of data collection was unknown, the year of publication of the article was used instead.

### 2.4. Scenario Analyses

#### 2.4.1. Iodine

In the current study, replacing salt with iodized salt was chosen as procedure for fortification of cooking aids with iodine. However, it should be noted that, in reality, for soy and fish sauces, it is recommended to add iodine as a separate fortificant at the end of the production process, because much of it will be lost during fermentation [19]. To determine the salt level in each product group (soy sauce, fish sauce and sweet soy sauce, flavored seasoning powders, and bouillon cubes), we used data from the USDA food composition table to have one standard (as local data were not always available). The salt content was estimated at 25% in fish sauce, 14% in soy sauce, 20% in fish and soy sauces combined, and 50% in flavored seasoning powders and bouillon cubes. 

The recommended iodine level in salt was set at 30 ppm (which is 30 µg of iodine per g of salt) based on a rough estimation of the average of local regulations published at the Global Food Fortification data website [5]. Because regulated levels of iodine in salt differed substantially between Southeast Asian countries, additional scenario analyses were performed with the lowest (18 ppm) and the highest (70 ppm) salt iodization levels. 

The potential impact of iodized salt on mean iodine intake was calculated as the average daily consumption of the product group multiplied by the salt content (as a percentage), multiplied by the iodine level of salt in the product. In addition, we calculated the relative contribution to the Codex NRV for adults (150 µg/day) [8].

#### 2.4.2. Iron

In the absence of guidelines or standards for fortification of condiments, seasonings and bouillon cubes with iron, the levels of iron for the scenario analyses were based on values reported in the literature [16,20,21,22,23]. A higher fortification level was chosen for seasonings and bouillon cubes because daily consumption levels were much lower compared to fish and soy sauce. For soy and fish sauces, scenarios included 0.1, 0.5, and 1.0 mg of iron per g of intake, respectively. For bouillon cubes and seasonings, scenarios included 0.5, 1.0, and 1.5 mg of iron per g of intake.

The potential impact of iron fortification on mean iron intake was calculated as the average daily (per capita) consumption of the products multiplied by the iron quantity per gram of the product. In addition, we calculated the relative contribution to the Codex NRV for adults (14 mg/day) [8].

## 3. Results

**Figure 1:**
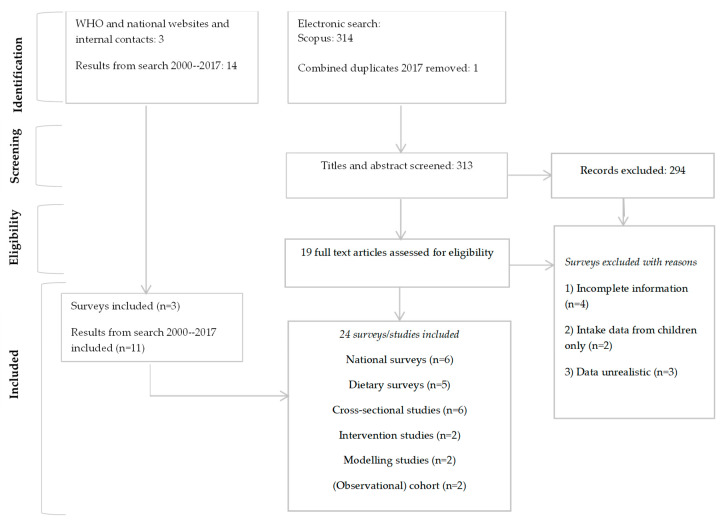
PRISMA flow diagram of the identification of literature for inclusion in this systematic search.

In total, 24 different studies were found that contained information on intake of soy sauce, fish sauce, bouillon cubes, seasonings, or other cooking aids in Asian countries. A PRISMA flowchart of the selection process is shown in Figure 1. Included sources covered cross-sectional studies, consumption surveys, data modeling studies, nutrition and health surveys, or randomized controlled efficacy trials. Because of unclear descriptions and unclarity about the composition of the products in categories defined as ‘other cooking aids’ category, we excluded the studies only reporting on this category. An overview of the 18 included studies and relevant characteristics is provided in Supplementary Table S1 [17,22,24,25,26,27,28,29,30,31,32,33,34,35,36,37,38,39]. Studies used different methods for data collection, e.g., food frequency questionnaires, (24 h) recalls, food weighing records, and interviews. Sample sizes of the studies varied from 26 participants to 75,000 participants.

### 3.1. Consumption Data of Condiments, Seasonings, and Bouillon Cubes

**Figure 2:**
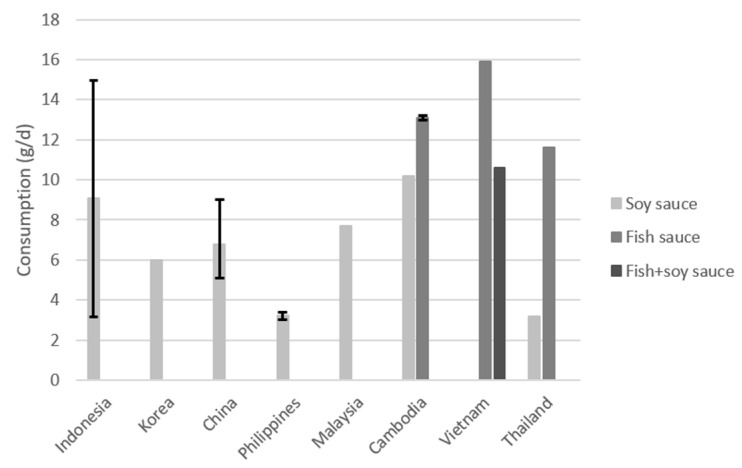
Average consumption of soy and fish sauce in g/day. The error bars represent the highest and lowest value found for the country.

Average intake of the condiments, soy and fish sauces, as reported in the included publications, is shown in Figure 2. For soy sauce, information on intake was available for six different countries. The data indicate that the mean consumption of soy sauce was highest in Cambodia (10.2 g/day) and lowest in the Philippines and Thailand (3.2 g/day). Fish sauce consumption was higher and only available for Cambodia (13 and 13.2 g/day), Vietnam (15.9 g/day), and Thailand (11.6 g/day). Another survey conducted in Vietnam showed a combined intake of soy and fish sauces of 10.6 g/day. 

**Figure 3:**
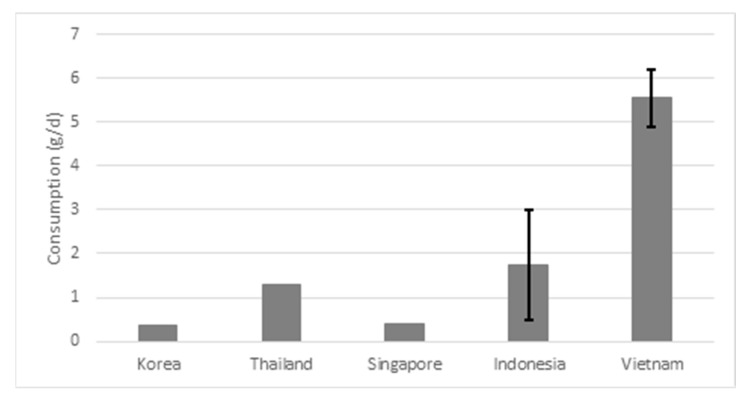
Average consumption of seasonings and bouillon cubes in g/day. The error bars represent the highest and lowest value found for the country.

Figure 3 shows the average daily consumption of seasonings and bouillon cubes. There was a high variation in intake between countries. For seasonings and bouillon cubes, intake ranged from 0.4 g/day in Korea and Singapore to 5.6 g/day in Vietnam.

### 3.2. Potential Impact of Iodized Salt on Iodine Intake

#### 3.2.1. Soy and Fish Sauces

**Figure 4:**
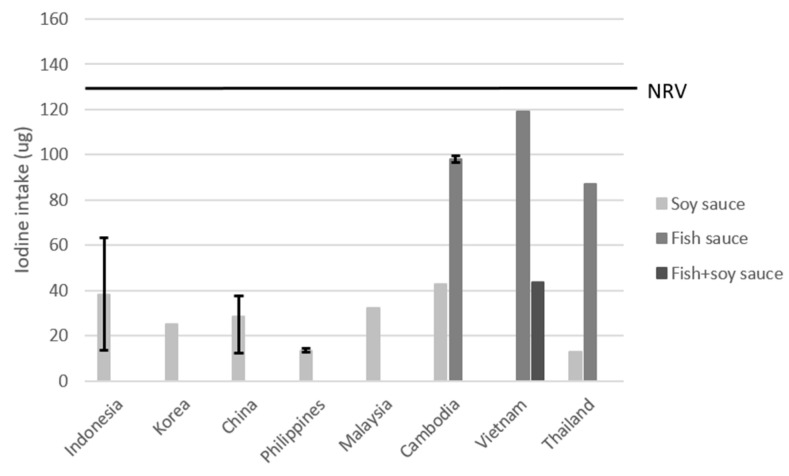
Estimated iodine intake after hypothetical fortification of soy and fish sauces with 30 µg of iodine/g of salt. The error bars represent the highest and lowest value found for the country. NRV = Codex nutrient reference value of 150 µg/day.

Figure 4 shows the results from the scenario using 30 µg of iodine per g of salt added for soy and fish sauces. Fortification of soy sauce with iodized salt would increase iodine intake by 14 to 43 µg per day. This would translate to 9–29% of the NRV being delivered by fortification. As the reported intake and salt level of fish sauce was higher, iodine intake from consuming fortified fish sauce in Thailand would deliver 58% of the NRV, 66% for Cambodia, and even 106% in Vietnam. The survey in which fish and soy sauces were combined showed that iodine fortification could deliver 42% of the NRV. Detailed results are provided in Supplementary Table S2.

As mentioned, additional scenarios were modelled using lower or higher fortification levels (see Table S2). When using a lower fortification level of 18 µg of iodine per g of salt, iodine intake was 8–26 µg for soy sauce (5–17% of the NRV), 52–72 µg for fish sauce (35–48% of the NRV), and 38 µg for fish and soy sauces combined (25% of the NRV). When a higher fortification level of 70 µg of iodine per g of salt was applied, iodine intake was 31–100 µg for soy sauces (21–67% of the NRV), 203–278 for fish sauces (135–186% of the NRV), and 148 µg for fish and soy sauces combined (99% of the NRV).

#### 3.2.2. Seasonings and Bouillon Cubes

**Figure 5:**
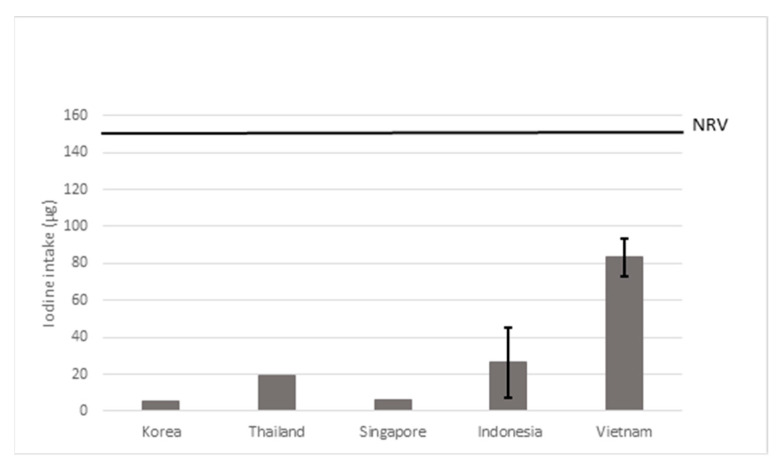
Estimated iodine intake after hypothetical fortification of seasonings and bouillon cubes with 30 µg of iodine/g of salt. The error bars represent the highest and lowest value found for the country. NRV = Codex nutrient reference value of 150 µg/day.

Figure 5 includes the results of the scenario using a fortification level of 30 µg of iodine/g of salt for seasonings and bouillon cubes. For both Korea and Singapore, fortification of seasonings or bouillon cubes would lead to an intake of 5 µg of iodine/day, which is 4% of the NRV, whereas in Vietnam the intake would be 83 µg of iodine/day contributing to 56% NRV. Detailed results are provided in Supplementary Table S3.

Additional scenarios with a lower fortification level (18 µg of iodine/g of salt) increased the iodine intake of bouillon cubes and seasonings by 3–50 µg (2–33% of the NRV). When a higher fortification level of 70 µg of iodine/g of salt was used, the iodine intake of seasonings and bouillon cubes increased to 13–194 µg (8–130% of the NRV) (see Table S3).

#### 3.2.3. Potential Impact or Iron Fortification on Iron Intake

##### Soy and Fish Sauces

**Figure 6:**
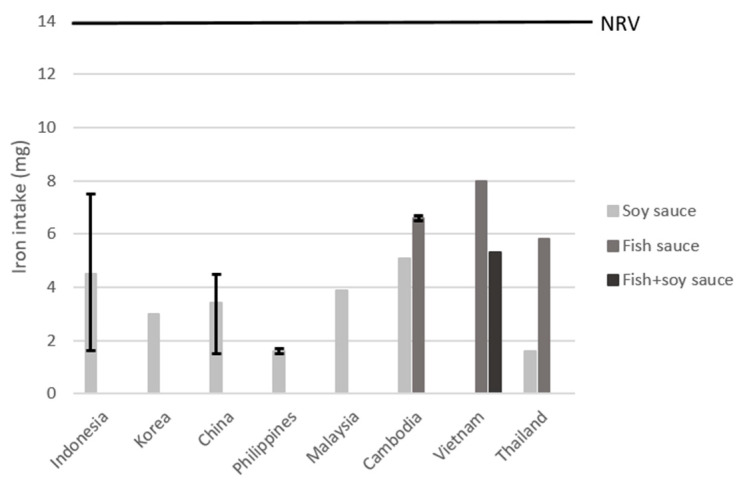
Estimated iron intake after hypothetical fortification of soy and fish sauces with iron fortification levels of 0.5 mg/g product. The error bars represent the highest and lowest value found for the country. NRV = Codex nutrient reference value of 14 mg/day.

In Figure 6, the scenario with a fortification level of 0.5 mg per g of food product is shown. Due to large variation in intake of soy sauce among Asian countries, iron fortification would result in an additional intake ranging from 1.6 mg/day (11% of the NRV) in the Philippines and Thailand to 4.5–5.1 mg/day (32–36% to the NRV) in Indonesia and Cambodia. Fish sauce consumption data from Cambodia, Vietnam, and Thailand showed that, after fortification, iron intake could be increased with 6.6, 8.0, and 5.8 mg per day (47%, 57%, and 41% of the NRV). Intake data on fish and soy sauces combined led to an iron intake of 5.3 mg for Vietnam (38% of the NRV). More detailed results can be found in Supplementary Table S4. 

Modeling with a lower fortification level of 0.1 mg of iron per g of food product did not lead to meaningful contributions to the NRV (all < 15%). A higher fortification level of 1 mg of iron per g of food product resulted in higher contribution to the NRV, which was 23–94% of the NRV for soy sauce, 83–114% of the NRV for fish sauce, and 76% of the NRV for soy and fish sauces combined (see Table S4).

##### Seasonings and Bouillon Cubes

**Figure 7:**
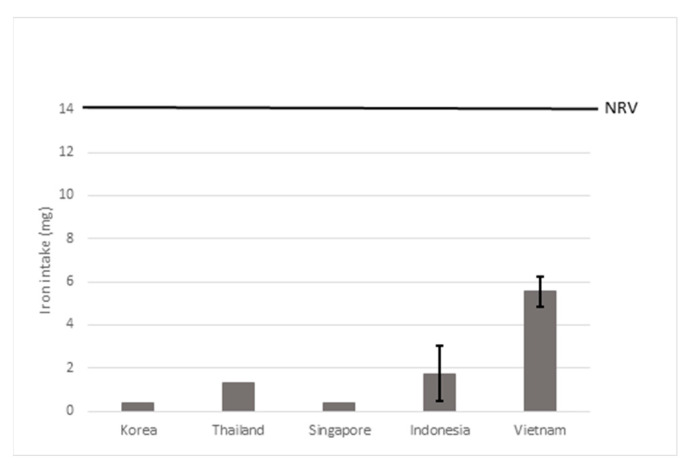
Estimated iron intake after hypothetical fortification of seasonings and bouillon cubes with iron fortification levels of 1 mg/g product. The error bars represent the highest and lowest value found for the country. NRV = Codex nutrient reference value of 14 mg/day.

The iron intake for seasonings and bouillon cubes after hypothetical fortification with 1 mg of iron per g of product is shown in Figure 7 and ranged from 0.4 mg/day (3% of the NRV) for Korea and Singapore to 5.6 mg/day (40% of the NRV) in Vietnam. More detailed information can be found in Supplementary Table S5.

Modeling with a lower fortification level of 0.5 mg of iron per g of food product) resulted in little extra iron intake in most countries of 0.2–0.9 mg (1–6% of the NRV), with the exception of Vietnam where iron intake would increase to 2.8 mg/day (20% of the NRV). Modeling with a higher fortification level of 1.5 mg of iron per g of food product) increased iron intake with 0.6–8.4 mg/d (4–59% of the NRV) (see Table S5).

## 4. Discussion

In this modeling study, we showed that adding iodized salt and iron fortification to soy and fish sauce, seasonings, and bouillon cubes could make a substantial contribution to the daily intake of iron and iodine, depending on the consumption of the different cooking aids. Mean consumption was 3.2–15.9 g/day for soy and fish sauces, and 0.4–5.6 g/day for seasonings and bouillon cubes. For soy and fish sauces, using iodized salt could help to increase the intake of iodine by 9–80% of the NRV, and iron fortification could increase iron intake by 11–57% of the NRV. For seasonings and bouillon cubes, using iodized salt could help to increase the intake of iodine by 4–56% of the NRV, and iron fortification could increase iron intake by 3–40% of the NRV.

Our study is the largest review on intakes of bouillons and seasonings in the Asian region. Moreover, it is the first study investigating the potential impact of both salt iodization and iron fortification and comparing the results of over the different countries in Southeast Asia and China. An important strength of the study is that data on intake and consumption of the food groups of interest were based on a systematic review. In addition, multiple scenarios with different iodine levels in salt and/or fortification levels were performed to explore the most optimal levels.

The study also had some limitations. It should be noted that modeling to predict the effect of fortification is a theoretical approach, and results are dependent on the quality of the input data and assumptions made. First, data on the consumption of condiments, seasonings, and bouillon cubes in Asian countries is rather limited and not harmonized in the way of reporting (e.g., in means, medians, per capita intake, or salt intake from the product). Therefore, data had to be converted, which may have led to inaccuracies of the data. Another limitation is related to the estimated salt content in the products for which we made the assumption that total sodium content of the product came from salt only. However, a small part of the sodium in the product may have originated from monosodium glutamate (MSG); hence, salt levels in the product and the consequent impact of iodized salt on iodine intake may have been slightly overestimated. Furthermore, we did not account for loss of iodine from iodized salt; however, limited available data indicate that losses from bouillon cubes and powder sachets would be around 13.6% and 0.8%, respectively [15].

In a similar study by Knowles et al. (2017) in Indonesia and the Philippines [17], the contribution of using iodized salt in cooking aids to iodine intake was lower compared to our findings for both countries [17]. This may have been due to lower consumption rates found for the cooking aids in the study of Knowles, as well as due to the lower salt percentages (e.g., for soy sauce in Indonesia). In fact, cooking aids of Unilever brands that are currently on the market in Asia have a lower salt content than the salt content of cooking aids in the USDA food composition table that was used for the scenario analyses in the current paper. This could imply that the effect on iodine intake found in this study may have been overestimated. However, despite these limitations, the results give a reasonable indication of the impact that fortification could have.

Salt is a well-known vehicle for iodization, and the WHO strongly recommends iodization of all salt in food, both table salt and salt added during food processing. The WHO also emphasizes that salt iodization and salt reduction should go hand-in-hand [40]. Reducing salt content in processed foods is a key strategy to lower sodium intake; therefore, reducing the amount of salt in condiments, seasonings, and bouillon cubes can help consumers to lower sodium intake. The effect of sodium-reduced bouillon cubes on sodium intake was evaluated in a study in South Africa where participants prepared dishes with regular versus sodium reduced bouillon cubes [41]. Over 80% of the participants did not add salt at the table to the dishes prepared with the sodium-reduced bouillon cubes to compensate for the lower sodium level. On average, sodium intake could be reduced up to 24% and both dishes were equally liked. However, as sodium reduction programs will also lower iodine intake from iodized salt, it remains important for public health authorities to keep monitoring iodine status and take appropriate actions to ensure a sufficient iodine supply in the diet by increasing the level of iodine in salt when needed. 

The current study showed a high variation between countries In terms of intake of the food groups considered. In particular, consumption data for the food group seasonings and bouillon cubes had a broad range of intake. A reason for this could be the cultural differences between countries, where seasonings and bouillon cubes may only be consumed on 1–2 days per week, whereas, in some countries such as Vietnam, consumption may be 5–7 times per week. Therefore, for iron, deciding on the appropriate fortification level is highly dependent on the consumption of the specific vehicle of interest in a country and requires local considerations to ensure effective fortification strategies. Moreover, the bioavailability of the iron compound chosen for fortification may vary and, therefore, levels may need to be adjusted [42]; however, the most soluble and bioavailable iron complexes can also cause the most organoleptic problems, such as precipitation and changes in color and flavor [43]. Similarly, the habitual diet of the population is known to have an impact on the iron absorption, where diets high in phytate from plant foods have an inhibiting effect. This is recognized in daily reference intakes and needs to be taken into account when setting the fortification levels [42]. Lastly, combining iron and iodine in one product may cause loss of iodine as has been shown with ferric pyrophosphate in double-fortified salt and not with encapsulated ferric fumarate [44]. However, the effect of iron fortification on iodine stability in cooking aids is not well studied. For iodine, the situation is different as, in most countries, the level of iodine in salt is regulated, and it would need to be adjusted upon national monitoring of iodine status. The Iodine Global Network has issued guidance for countries to assess the contribution of iodized salt in processed foods to population iodine status, and whether action is needed to achieve or sustain this [45]. In addition, it is important to evaluate equity in terms of accessibility to and health benefits of fortification strategies for different population groups within countries [46].

Safety is a key element for fortification. The scenario analyses showed that the intake of both micronutrients after fortification would still be below the NRV for all countries. However, in reality, the intake of iodine and iron also comes from other sources in the diet, such as fish for iodine, and meat, whole grains, nuts, and vegetables for iron. Unfortunately, hardly any reliable quantitative data exist on the iodine and iron intake and their dietary sources for Asian countries. Food composition tables often lack data on iodine content, and use of (iodized) table salt is generally not covered in food consumption surveys. This makes it difficult to interpret our findings in perspective of the total diet. Nevertheless, no safety issues are expected, which can be illustrated by an example based on the limited available data from China. In China, average daily intake of iron is 21.4 mg/day [47]. After fortification with the highest fortification level (1 mg/g food product for soy sauce [48]), the total iron intake would increase to 30.4 mg/day, which is still well below the UL of 45 mg/day. For use of iodized salt in processed foods, China employs different regulations per province to mitigate the risk of iodine intakes exceeding the upper level [49]. Unfortunately, we did not have any data available on total iodine intake for China. However, while the scenario of 30 ppm is slightly higher than the maximal 26 ppm allowed in the regions with the regulated lowest iodine levels in salt [50], the contribution to iodine intake would be relatively modest with 28 µg/day (19% of the NRV). In addition, the modeling study from Thailand showed that, with use of iodized salt in multiple processed foods, as well as household salt, iodine intakes would be maximally 230 µg/day and stay well below the UL for iodine of 600 µg/day [16]. 

The current study shows that the food industry can have a positive impact on health by improving micronutrient intake through food fortification and by replacing salt with iodized salt in food products, such as cooking aids, where needed. In line with like-minded companies, Unilever has taken up this opportunity; in 2022, the company achieved its commitment to provide 200 billion servings of products with a meaningful amount (≥15% NRV per serving) of iron, iodine, zinc, and vitamins A and D. Moreover, a new commitment was set to double the number of products with positive nutrients and ingredients across the portfolio by 2025 [51]. In this way, food companies can help people get the nutrients they need.

To create a more global perspective, the scenario analyses applied in this study could be repeated for different regions. However, to ensure valid results, future research should focus on generating nationally representative and more recent data on intake of food groups such as seasonings, bouillon cubes, and condiments (including soy and fish sauces), with clear definitions for the products included in the food groups, especially for condiments. In addition, there is a clear need for data on dietary intake of micronutrients in order to define effective and safe fortification levels for foods.

## 5. Conclusions

In conclusion, the scenario analyses performed in this study showed that fortification of condiments, seasonings, and bouillon cubes with iodine and iron can provide a substantial contribution to the daily intake of these micronutrients. Cooking aids are generally widely consumed in populations, coming from different socioeconomic classes and both rural and urban areas, which makes them suitable for a role in increasing the micronutrient intake by fortification. However, consumption of different cooking aids varies highly across countries, which needs to be taken into consideration when deciding on the right vehicle and level of fortification if not regulated as in the case of salt iodization. 

## Data Availability

Not applicable.

## Supplementary Materials

The following supporting information can be downloaded at https://www.mdpi.com/article/10.3390/nu15030616/s1: Table S1. Overview of included studies; Table S2. Iodine intake following fortification with different iodine levels in iodized salt for soy and fish sauces; Table S3. Iodine intake following fortification with different iodine levels in iodized salt for seasonings and bouillon cubes; Table S4. Iron intake following fortification with different iron levels for soy and fish sauces; Table S5. Iron intake following fortification with different iron levels for seasonings and bouillon cubes.
