# Supplementary material for: The Potential of Condiments, Seasonings, and Bouillon Cubes to Deliver Essential Micronutrients in Asia: Scenario Analyses of Iodine and Iron Fortification
Source: Nutrients. 2023 Jan 25;15(3):616. doi: 10.3390/nu15030616 (PMC9920952; doi:10.3390/nu15030616)
Supplement: Supplementary file 1 [file nutrients-15-00616-s001.zip › nutrients-2106077-supplementary.pdf]

Supplementary materials

Table S1. Overview of included studies

Reference	Year	Country	Region	Characteristic Study	Age Group	Gender	Sample Size	Methodology	Food Group
**Andarwulan [1]**	2011	Indonesia	Jakarta (urban)	Cross-sectional study	All	M+F	222 households	Questionnaire on condiments and seasoning usage	Soy sauce
		Indonesia	Bogor (rural)	Cross-sectional study	All	M+F	222 households	Questionnaire on condiments and seasoning usage	Soy sauce
**Choi [2,3]**	2020	Korea	Seoul, Chungcheong-do, Gyeongsang-do, Jeolla-do	Evaluation of nutrient intake of Mn with use of dietary intake survey	Adults	M+F	640	24-h recall, executed together with dietitian	Soy sauce, seasoning
**Chotivichien [4]**	2021	Thailand	National data	Data modelling based on consumption data and food labelling/laboratory analysis	Non-pregnant adults	M+F (non-pregnant)	NR	National sodium chloride intake survey and market share reports	Seasoning powder, soy sauce, fish sauce
**Choy [5]**	2021	Singapore	Nationwide	Cross-sectional	6-12	M+F	561	24-h recall, 2 times (one weekend and one weekday)	Bouillon cubes/flavourings, essence, yeast extracts
**Do [6]**	2014	Vietnam	Rural	Survey on sodium intake	Adults	M+F	121	24hr direct weighing	Seasoning, fish sauce
**Fang [7]**	2020	China	Nationwide	China National Nutrition and Health Surveillance CNNHS 2010-2012	Adults	M+F	53993	24-h recall, 3 times, combined with household weighing method. Done by dietary trained staff. Sodium intake calculated with Chinese FCT	Soy sauce

**Knowles [8]**	2017	Indonesia	Nationwide	Data modelling based on market data	-	-	-	GAIN analysed data from Euromonitor passport 2013 and PT Neilson company	Soy sauce, stock (including seasoning)
		Philippines	Nationwide	Data modelling based on market data	-	-	-	2008 National Nutrition Survey (NNS) chapter on household food consumption	Soy sauce
**Lauillou [9]**	2012	Vietnam	national	Food consumption survey	19-50y	F	7680 households	24h recall with controlled food weighing	Soy sauce, fish sauce, flavouring powder
**Lee [10]**	2005	Philippines	Metropolitan Cebu	Cebu Longitudinal Health and Nutrition Survey - cohort	Adults	F	1776	Two 24-hour dietary recalls + semi structured questionnaire	Soy sauce, fish sauce
**Malaysia [11]**	2014	Malaysia	National	Malaysian Adult Nutrition Survey (MANS)	Adults 18-59y	M+F	NR	24h recall and FFQ	Soy sauce
**Philippines national intake data [12]**	2015	Philippines	National	Dietary survey	All	M+F	9930 households	Food weighing, food inventory, food recall	Soy sauce,
**Theary [13]**	2012	Cambodia	National	Cross-sectional survey	Adults	M+F	2797 households	Questionnaire on knowledge and utilization of soy and fish sauce	Soy sauce, fish sauce
**Whitfield [14]**	2016	Cambodia	PreyVeng Province (rural areas)	Community-based, double-blind, 3 parallel-arm randomized controlled efficacy trial	Adults	M+F	276	3-day weigh fish sauce record	Fish sauce
**Yu [15]**	2018	China	Nationwide	China health and nutrition survey	Adults	M+F	15510	Daily record	Soy sauce
**Yu [16]**	2011	China	National	China health and nutrition survey	Adults	M+F	12.344	Soy sauce consumption was determined on a daily basis by calculating changes in home food stock	Soy sauce
**Zhai [17]**	2005	China	132 counties in 31 provinces	Cross-sectional survey	Adults	M+F	68.962	24 h recall on 3 consecutive days and condiments data collected with weighing method	Soy sauce

**Zhu [18]**	2019	China	Shanghai	Observational cohort	>15 y	M+F	1704	3-d 24-h recall + household weighing. Chinese Database used to define groups.	Soy sauce

Table S2. Iodine intake following fortification with different iodine levels in iodized salt for soy and fish sauces.

Country	Type of Condiment	Mean Intake of Condiment (g/d)	Assumed Salt Content of Condiment (%)	Mean Salt Intake from Condiment g/d)	Mean Iodine Intake following Fortification with Iodized Salt at Different Levels					
					18 ppm		30 ppm		70 ppm	
					µg/d	%NRV *	µg/d	%NRV *	µg/d	%NRV *
**Indonesia [1,8]**	soy sauce	9.1	14	1.3	23	15	38	25	89	59
**Korea [2]**	soy sauce	6.0	14	0.8	15	10	25	17	59	39
**China [7,15–18]**	soy sauce	6.8	14	0.9	17	11	28	19	66	44
**Philippines [10,12]**	soy sauce	3.2	14	0.4	8	5	13	9	31	21
**Malaysia [11]**	soy sauce	7.7	14	1.1	19	13	32	22	75	50
**Cambodia [13]**	soy sauce	10.2	14	1.4	26	17	43	29	100	67
**Cambodia [13,14]**	fish sauce	13.1	25	3.3	59	39	98	66	229	153
**Vietnam [6]**	fish sauce	15.9	25	4.0	27	18	119	80	104	69
**Vietnam [9]**	fish+soy sauce	10.6	20	2.1	72	48	64	42	278	186
**Thailand [4]**	fish+soy sauce	7.4	20	1.5	38	25	44	30	148	99

* NRV = Codex nutrient reference value, 150 µg/d for iodine

Table S3. Iodine intake following fortification with different iodine levels in iodized salt for seasonings and bouillon cubes.

Country	Type of Cooking Aid	Mean Intake of Cooking Aid (g/d)	Assumed Salt Content of Cooking Aid (%)	Mean Salt Intake from Cooking Aid g/d)	Mean Iodine Intake following Fortification with Iodized Salt at Different Levels					
					18 ppm		30 ppm		70 ppm	
					µg/d	%NRV *	µg/d	%NRV *	µg/d	%NRV *
**Korea [3]**	seasoning	0.4	50	0.2	3	2	5	4	13	8
**Thailand [4]**	seasoning powder	1.3	50	0.7	12	8	20	13	46	30
**Singapore [5]**	bouillon cubes/flavourings, essence, yeast extracts	0.4	50	0.2	4	2	6	4	14	9
**Indonesia [31]**	seasoning/stock	1.8	50	0.9	16	11	26	18	61	41
**Vietnam [6,9]**	seasoning/flavouring powder	5.6	50	2.8	50	33	83	56	194	130

* NRV = Codex nutrient reference value, 150 µg/d for iodine

Table S4. Iron intake following fortification with different iron levels for soy and fish sauces.

Country	Type of Condiment	Mean Intake of Condiment (g/d)	Mean Iron Intake following Fortification with Different Iron Levels					
			0.1 mg Iron / g Product		0.5 mg Iron / g Product		1 mg Iron / g Product	
			mg/d	%NRV *	mg/d	%NRV *	mg/d	%NRV *
**Indonesia [1,8]**	soy sauce	9.1	0.9	6	4.5	32	9.1	64
**Korea [2]**	soy sauce	6.0	0.6	4	3.0	21	6.0	42
**China [7,15–18]**	soy sauce	6.8	0.7	5	3.4	24	6.8	48
**Philippines [10,12]**	soy sauce	3.2	0.3	2	1.6	11	3.2	22
**Malaysia [11]**	soy sauce	7.7	0.8	6	3.9	28	7.7	56
**Cambodia [13]**	soy sauce	10.2	1.0	7	5.1	36	10.2	72
**Cambodia [13,14]**	fish sauce	13.1	1.3	9	6.6	47	13.1	94
**Vietnam [6]**	fish sauce	15.9	1.6	5	8.0	57	15.9	114
**Vietnam [9]**	fish+soy sauce	10.6	1.1	11	5.3	38	10.6	76
**Thailand [4]**	fish+soy sauce	7.4	0.7	8	3.7	26	7.4	52

* NRV = Codex nutrient reference value, 14 mg/d for iron

Table S5. Iron intake following fortification with different iron levels for seasonings and bouillon cubes.

Country	Type of Cooking Aid	Mean Intake of Cooking Aid (g/d)	Mean Iron Intake following Fortification with Different Iron Levels					
			0.5 mg Iron / g Product		1 mg Iron / g Product		1.5 mg Iron / g Product	
			mg/d	%NRV *	mg/d	%NRV *	mg/d	%NRV *
**Korea [3]**	seasoning	0.4	0.2	1	0.4	3	0.5	4
**Thailand [4]**	seasoning powder	1.3	0.7	5	1.3	9	2.0	14
**Singapore [5]**	bouillon cubes/flavourings, essence, yeast extracts	0.4	0.2	1	0.4	3	0.6	4
**Indonesia [8]**	seasoning/stock	1.8	0.9	6	1.8	13	2.6	19
**Vietnam [6,9]**	seasoning/flavouring powder	5.6	2.8	20	5.6	40	8.3	59

* NRV = Codex nutrient reference value, 14 mg/d for iron

References

1. Andarwulan, N., et al., *Free Glutamate Content of Condiment and Seasonings and Their Intake in Bogor and Jakarta, Indonesia.* Food and Nutrition Sciences, 2011. **02**(07): p. 764-769.
2. Choi, M.K. and Y.J. Bae, *Dietary Intake and Urinary Excretion of Manganese in Korean Healthy Adults.* Biological Trace Element Research, 2020. **196**(2): p. 384-392.
3. Choi, M.K., et al., *Contribution of food groups to sodium and potassium intakes by their ratio in Korean adults.* Progress in Nutrition, 2019. **21**(2): p. 285-293.
4. Chotivichien, S., et al., *Assessment of the contribution of industrially processed foods to salt and iodine intake in Thailand.* PLoS ONE, 2021. **16**(7 July).
5. Choy, M.J.Y., I. Brownlee, and A.M. Murphy, *Data-driven dietary patterns, nutrient intake and body weight status in a cross-section of singaporean children aged 6–12 years.* Nutrients, 2021. **13**(4).
6. Do, H.T.P., *Hypertension in Vietnam:prevalence, risk groups and effects of salt substitution*, in *Nutrition and Disease, Human nutrition & Health, VLAG*. 2014, Wageningen University: Wageningen. p. 160.
7. Fang, K., et al., *Dietary sodium intake and food sources among Chinese adults: Data from the CNNHS 2010–2012.* Nutrients, 2020. **12**(2).
8. Knowles, J., et al., *Iodine intake through processed food: Case studies from Egypt, Indonesia, the Philippines, the Russian federation and Ukraine, 2010–2015.* Nutrients, 2017. **9**(8).
9. Laillou, A., et al., *Improvement of the Vietnamese diet for women of reproductive age by micronutrient fortification of staples foods and condiments.* PLoS One, 2012. **7**(11): p. e50538.
10. Lee, N.R., *Estimating the effects of overweight duration, sodium intake and genetic variants on hypertension risk among Filipino women in Cebu, Philippines*. 2009, The University of North Carolina at Chapel Hill.
11. Ahmad, M.H.B. *Key Findings of Recent Food Consumption and Nutrition Surveys in ASEAN - Malaysian Adult Nutrition Survey (MANS)2014*. in *symposium on dietary intakes*. 2016. Singapore.
12. (DOST-FNRI), D.o.S.a.T.-F.a.N.R.I., *Philippine Nutrition Facts and Figures 2015: Dietary Survey.* 2016: FNRI Bldg., DOST Compound, Gen. Santos Avenue, Bicutan, Taguig City, Metro Manila, Philippines. .
13. Theary, C., et al., *Fish sauce, soy sauce, and vegetable oil fortification in Cambodia: where do we stand to date?* Food Nutr Bull, 2013. **34**(2 Suppl): p. S62-71.
14. Whitfield, K.C., et al., *Household Consumption of Thiamin-Fortified Fish Sauce Increases Erythrocyte Thiamin Concentrations among Rural Cambodian Women and Their Children Younger Than 5 Years of Age: A Randomized Controlled Efficacy Trial.* Journal of Pediatrics, 2017. **181**: p. 242-247.e2.
15. Yu, L., et al., *Secular trends in salt and soy sauce intake among Chinese adults, 1997–2011.* International Journal of Food Sciences and Nutrition, 2018. **69**(2): p. 215-222.
16. Yu, D., et al., *Salt intake among Chinese adults in 2010-2012.* Zhonghua yu fang yi xue za zhi [Chinese journal of preventive medicine], 2016. **50**(3): p. 217-220.

17. Zhai, F.Y., et al., *Study on the current status and trend of food consumption among Chinese population.* Zhonghua liu xing bing xue za zhi = Zhonghua liuxingbingxue zazhi, 2005. **26**(7): p. 485-488.
18. Zhu, Z., et al., *The dietary intake and its features across four seasons in the metropolis of China.* Journal of Nutritional Science and Vitaminology, 2019. **65**(1): p. 52-59.
